# Supplementary material for: The role of government policy, social infrastructure and Fengshui in intending to buy tourism real estate
Source: PLoS One. 2023 Jul 27;18(7):e0281436. doi: 10.1371/journal.pone.0281436 (PMC10374126; doi:10.1371/journal.pone.0281436)
Supplement: S1 Table — (DOCX) [file pone.0281436.s001.docx]

**S1 Table.** Feng Shui factors for the surrounding environment element

| **Surrounding**  **environment** | **Feng Shui factors** | **Feng Shui perspective** | **Sources** |
| --- | --- | --- | --- |
| **Topography** | ﻿On top of hill or elevated hills | Unfavorable | [24] |
|  | ﻿Located on a low-lying location that is surrounded by hills | Unfavorable | [44] |
|  | The land on which the house is built is higher in the front and lower in the back | Unfavorable | [44] |
| **Front of site** | ﻿Facing cemetery | Unfavorable | [4] |
|  | Facing hospital | Unfavorable | [45] |
|  | Facing a distance between two tall towers | Unfavorable | [44] |
|  | Y-junction or T-junction | Unfavorable | [45] |
| **Rear of site** | River at back | Unfavorable | [46] |
|  | Mountain behind | Favorable | [47] |
| **Sides of site** | Beside electric substation | Unfavorable | [45] |
| **Street location** | Dead end | Unfavorable | [44] |
| **Water view** | Sea view | Favorable | [48] |
| **Wind direction** | Lack of wind |  |  |
|  | Strong wind | Unfavorable | [25] |
